# A synonymous mutation in *PI4KA* impacts the transcription and translation process of gene expression

**DOI:** 10.3389/fimmu.2022.987666

**Published:** 2022-10-19

**Authors:** Kaihui Zhang, Lili Kang, Haozheng Zhang, Lu Bai, Huanping Pang, Qinghua Liu, Xinyi Zhang, Dong Chen, Haihua Yu, Yuqiang Lv, Min Gao, Yi Liu, Zhongtao Gai, Dong Wang, Xiaoying Li

**Affiliations:** ^1^ Pediatric Research Institute, Children’s Hospital Affiliated to Shandong University, Jinan, China; ^2^ Department of Neonatology, Children’s Hospital Affiliated to Shandong University, Jinan, China; ^3^ Department of Pediatrics, The Second Hospital of Shandong University, Jinan, China; ^4^ Department of Ultrasonic imaging, Children’s Hospital Affiliated to Shandong University, Jinan, China; ^5^ Intensive Care Unit, The Second People’s Hospital of Shandong Province, Jinan, China

**Keywords:** *PI4KA*, diarrheal, immunodeficiency, synonymous mutation, minigene splicing assay

## Abstract

Phosphatidylinositol-4-kinase alpha (PI4KIIIα), encoded by the *PI4KA* gene, can synthesize phosphatidylinositol-4-phosphate (PI-4-P), which serves as a specific membrane marker and is instrumental in signal transduction. *PI4KA* mutations can cause autosomal recessive diseases involving neurological, intestinal, and immunological conditions (OMIM:619621, 616531, 619708). We detected sepsis, severe diarrhea, and decreased immunoglobulin levels in one neonate. Two novel compound heterozygous mutations, c.5846T>C (p.Leu1949Pro) and c.3453C>T (p.Gly1151=), were identified in the neonate from the father and the mother, respectively. Sanger sequencing and reverse transcription polymerase chain reaction (RT-PCR) for peripheral blood and minigene splicing assays showed a deletion of five bases (GTGAG) with the c.3453C>T variant at the mRNA level, which could result in a truncated protein (p.Gly1151GlyfsTer17). The missense mutation c.5846T>C (p.Leu1949Pro) kinase activity was measured, and little or no catalytic activity was detected. According to the clinical characteristics and gene mutations with functional verification, our pediatricians diagnosed the child with a combined immunodeficiency and intestinal disorder close to gastrointestinal defects and immunodeficiency syndrome 2 (GIDID2; OMIM: 619708). Medicines such as immunomodulators are prescribed to balance immune dysregulation. This study is the first report of a synonymous mutation in the *PI4KA* gene that influences alternative splicing. Our findings expand the mutation spectrum leading to PI4KIIIa deficiency-related diseases and provide exact information for genetic counseling.

## Introduction

**Figure 1:**
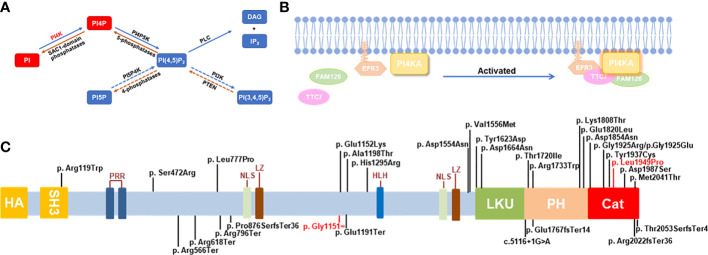
Schematic diagram for the role and mutations of *PI4KA*. **(A)** The pathway of phosphoinositide synthesis and degradation. Dashed arrows: in mammals only, phosphatidylinositol (PI), phosphatidylinositol-4-kinase (PI4K), messengers-diacylglycerol (DAG), inositol-1,4,5-triphosphate (IP3). **(B)** Schematic diagram of *PI4KA* interaction. **(C)** Schematic diagram of *PI4KA* domains and landscape of pathogenic variants. The putative *PI4KA* contains Src homology domain 3 (SH3; 81-132aa), proline-rich regions (PRR; 240-244aa and 266-276aa), nuclear localization signal (NLS; 980-984aa and 1473-1489aa), leucine zipper (LZ; 993-1021aa), helix–loop–helix (HLH; 1351-1364aa), lipid kinase unique domain (LKU; 1621-1689aa), Pleckstrin homology domain (PH; 1718-1851aa), and catalytic domain (Cat; 1848-2103aa). The 30 pathogenic variants of *PI4KA* reported are shown over the diagram, and the variant identified in this study is in red.

Phosphatidylinositol-4-kinase alpha (PI4KIIIα, NP_477352.3) is encoded by the *PI4KA* gene (NM_058004.4), which is located in chromosome 22q11.21 and comprised 55 exons. PI4KIIIα can synthesize phosphatidylinositol-4-phosphate (PI-4-P), a type of phosphoinositide (PIP) that serves as a specific membrane marker and is instrumental in signal transduction by generating two downstream hydrolysates as second messengers, diacylglycerol (DAG) and inositol1,4,5-triphosphate (IP3) (1) (**Figure 1A**). PI-4-Ps are the main and essential lipid determinants of the plasma membrane for the endosomal system, the Golgi complex, and the trans-Golgi network (TGN) and determine the acidic character of the plasma membrane (2, 3). In mammals, there are four PI4Ks that phosphorylate PI at position 4 of the inositol ring: PI4K IIα, IIβ, IIIα, and IIIβ. Type II and III *PI4KA* enzymes belonging to divergent sequence families have exhibited very different biochemical properties; for example, type II enzymes can be inhibited by adenosine but not type III isoform inhibitors, and type III enzymes are larger proteins and are sensitive to PI3K inhibitors, such as wortmannin and LY294002, because PI4KIII shows a great degree of similarity with the PI3K/protein kinase superfamily (4, 5). PI4KIIα is the most active PI4K in mammals, synthesizing approximately 50% of PI-4-P; however, inactive PI4KIIα, such as PI4K IIβ and IIIβ, rarely causes dangerous diseases in humans (2, 6). Furthermore, the functional abnormality of PI4KIIIα can cause severe and even lethal symptoms, including polymicrogyria, perisylvian, cerebellar hypoplasia, arthrogryposis (OMIM: 616531), spastic paraplegia 84, autosomal recessive paraplegia (OMIM: 619621), and gastrointestinal defects and immunodeficiency syndrome 2 (OMIM: 619708), in humans and gene disruptions that result in embryonic lethality in mice (7).

Perisylvian polymicrogyria with cerebellar hypoplasia and arthrogryposis (8, 9) (OMIM: 616531) is a severe autosomal recessive disorder characterized by global developmental delay with impaired intellectual development and poor or absent speech, axial hypotonia, and peripheral spasticity and hyperreflexia. Affected individuals can have feeding difficulties with gastroesophageal reflux and poor overall growth, as well as microcephaly and nonspecific dysmorphic facial features. Additional features may include nystagmus, inability to walk, ataxia, abnormal movements, and seizures. Brain imaging shows hypomyelination with decreased white matter volume, cerebral and cerebellar atrophy, and thin corpus callosum. Autosomal recessive spastic paraplegia–84 (OMIM: 619621) (8, 9) is characterized by an onset of slowly progressive walking difficulties due to lower limb weakness, stiffness, and spasticity in the first two decades of life. Some patients may have additional features including nystagmus, urinary urgency, joint contractures, and possible learning disabilities. Gastrointestinal defects and immunodeficiency syndrome 2 (OMIM: 619708) (9, 10) is a severe autosomal recessive developmental disorder characterized by multiple intestinal atresia apparent soon after birth. Affected infants have a distended abdomen and do not pass meconium. Some patients have some evidence of inflammatory bowel disease and have immunodeficiency such as T-, B-, and NK-cell lymphopenia and agammaglobulinemia.

PI4KIIIα is a very large protein of about 240 kDa and is ubiquitously distributed in human tissues, particularly in the brain and placenta (11), with little or no expression in the lung, liver, pancreas, testis, or leukocytes. In the cell, PI4KIIIα is predominantly distributed in the endoplasmic reticulum (ER) and is also detected in the pericentriolar Golgi region, surrounding the mitochondrial region, multivesicular bodies, and the nucleolus. The endogenous PI4KIIIα located on these organelle membranes, representing some form of inter-organelle junction, is speculated to permit the communication of PI-4-P or PI4KIIIα proteins between closely juxtaposed compartments (12, 13).

PI4KIIIα forms a stable, homodimeric ~700-kDa lipid kinase complex with two regulatory subunits, TTC7 and FAM126, in human cells, each of which exists as two isoforms, A and B (14–20), respectively, and has overlapping functions but different tissue distributions (14, 15, 17–20). TTC7 can stabilize the PI4KIIIα complex, and mutations in *TTC7A*, a TTC7 homolog, have been associated with a rare hereditary human disease, combined immunodeficiency with multiple intestinal atresia (CID-MIA) (OMIM: 609332), also known as gastrointestinal defects and immunodeficiency syndrome (GIDID). To date, TTC7A and PI4KIIIα variants are the only known genetic causes of MIA in humans (9, 21), and the *FAM126A* variants may cause leukodystrophy hypomyelinating 5 (OMIM:610532) with the clinical features of nystagmus, ataxia, and spasticity with or without peripheral neuropathy (22), similar to the neurological symptoms caused by *PI4KA* mutations. Thus, the PI4KIIIα complex plays an important role in intestinal and brain development, including myelination, which can explain the clinical features of abnormal PI4KIIIα.

## Patients

The propositus, a girl weighing 2.7 kg, was prematurely delivered at 34 + ^6^ weeks by vaginal birth; the amniotic fluid was 2 degrees polluted, and the umbilical cord was wrapped around the neck. She was the firstborn of her mother, who had hypothyroidism due to taking left thyroid medicine to balance the hormone level, and her physical condition was fine during pregnancy. The family is a non-consanguineous marriage, and the parents are both 29 years old and healthy. The birth process and the condition of the fetus were good. There was an onset of diarrhea with yellow loose stool more than 10 times daily and a milk flap at 6 days after birth. The 20-day-old neonate was hospitalized for treatment. Medicines, fluid infusion, antibiotics, smectite powder, probiotics, etc., were applied for infection and diarrhea. However, the condition of the newborn worsened, accompanied by fever, poor reaction, and feeding, and the number of white cells was up to 45.04*10^9^/L with infection by *Staphylococcus epidermidis* and *Enterococcus faecium*, which were found by blood and sputum culture, respectively. Therefore, the neonate was transferred to the neonatal intensive care unit (NICU) of our hospital at 26 days old with a diagnosis of neonatal sepsis and severe diarrhea (persistence). A detailed physical examination was performed (temperature =36.7°C, breathing =152 times/min, weight =1.99 kg, blood pressure =84/35 mmHg, height =46 cm, head circumference =32 cm), which revealed immature and malnourished development. No neurological symptoms and physical deformity in appearance were found other than hypotonia in the extremities and left hand Tongguan palm. Our pediatrician suspected that the neonate may have immunodeficiency disease based on the clinical features of persistent diarrhea, fever, and progressive increase in white blood cells after anti-infective therapy. Neonatal immunodeficiency is mostly caused by monogenic genetic diseases. Therefore, screening for immune function and whole exome sequencing were applied for pathogenesis.

### Routine tests, metabolic analysis, and gastrointestinal pathology

Exhaustive laboratory examinations were performed on the propositus. At the acute phase, the white cell count was up to 29.31 (3.5–9.5) *10^9^/L, among which the neutrophil, eosinophilic, and basophilic granulocytes increased the most, with values of 20.04 (1.8–6.3)*10^9^/L, 3.78 (0.02–0.52)*10^9^/L, and 0.16 (0–0.06)*10^9^/L, respectively. Procalcitonin (0.543 ng/ml) and C-reactive protein (14.66 mg/L) levels increased, indicating infection in the body. Immunoglobulins (Ig) were detected with elevated IgA levels of 0.07 (0.01–0.06] g/L) and descended IgG levels of 1.27 (6.6–17.5 g/L) and IgM levels of 0.04 (0.06–0.2) g/L, all of which showed abnormal humoral immunity. Routine cerebrospinal fluid tests, including glucose, cells, protein quantification, chlorine, and bacteria, were all normal. The stool sample was examined, in which fecal occult blood tests were positive, and white cells, pyocytes, and red cells were all strongly positive. The levels of blood transaminase, dehydrogenase, ammonia, and glucose were all normal, indicating normal functioning of the liver and kidney. The blood levels of protein, enzymes, and lipids were decreased. The total protein was 31.8 (60–80) g/L, including albumin, 18.9 (35–52) g/L; globulin, 12.9 (20–30) g/L; amylase, 12 (15–121) U/L; amylopsin, <1.0 (7–115) U/L; lipase, 8 (13–60) U/L; and total cholesterol, 2.88 (3.15–6.2) mmol/L. Blood electrolyte levels were disturbed, chlorine was raised to 109 (96–108) mmol/L, and carbon dioxide decreased to 17.1 (22–28) mmol/L.

Blood carnitine and amino acids from dried blood spots were screened by liquid chromatography–tandem mass spectrometry (LC-MS/MS) using an Applied Biosystems API 3200 analyzer (ABSCIEX, Foster City, USA) and ChemoView software (ABSCIEX, Foster City, USA). The organic acids were measured by gas chromatography–mass spectrometry (GC/MS) using a GCMS-QP2010 analyzer (Shimadzu, Tokyo, Japan) and Inborn Errors of Metabolism Screening System software (Shimadzu, Tokyo, Japan). None of the two screenings were unusual, thus eliminating the possibility of common genetic metabolic diseases.

The kappa-deleting recombination excision circle (KREC) detected by specific real-time PCR had decreased, while the t-cell receptor excision circle (TREC) still remained normal, which suggested that the B lymphocytes were possibly insufficient or dysfunctional. Meanwhile, T lymphocyte subgroups were detected by flow cytometry, and the proportions of total T lymphocytes (CD3+, 85.73%) and helper T lymphocytes (CD3+ CD4+, 68.36%) were normal. However, the proportion of cytotoxic T lymphocytes (CD3+ CD8+, 14.07%) decreased slightly, and the ratio (CD4+/CD8+, 4.86) increased.

No typical gastrointestinal pathological change was detected for inflammatory bowel disease. In addition, the gastric mucosa showed that the foveal epithelium fell off, there was no gastric fundus gland, there were a few pyloric adenoid glands, there were scattered lymphocytes and eosinophils, and the duodenal mucosa had no clear villi structure, interstitial edema, or scattered lymphocyte infiltration. The terminal ileum presented no villus structure, significantly proliferated crypt epithelium, visible epithelial apoptosis, and many eosinophils (>100/HPF), and some lymphocytes infiltrated the stroma. The ascending colon and rectal mucosa showed significant hyperplasia of the crypt epithelium with apoptosis, expansion of the interstitial volume in small vessels, scattered eosinophils (~50/HPF), and a small amount of lymphocyte infiltration. The transverse colon revealed inflammatory exudate, a small amount of broken intestinal epithelium, many eosinophils, and obvious hyperplasia of intestinal epithelial cells, with occasional apoptosis. All the above results suggest mild inflammation of the gastrointestinal tract.

### Ultrasonic Doppler examination

**Figure 2:**
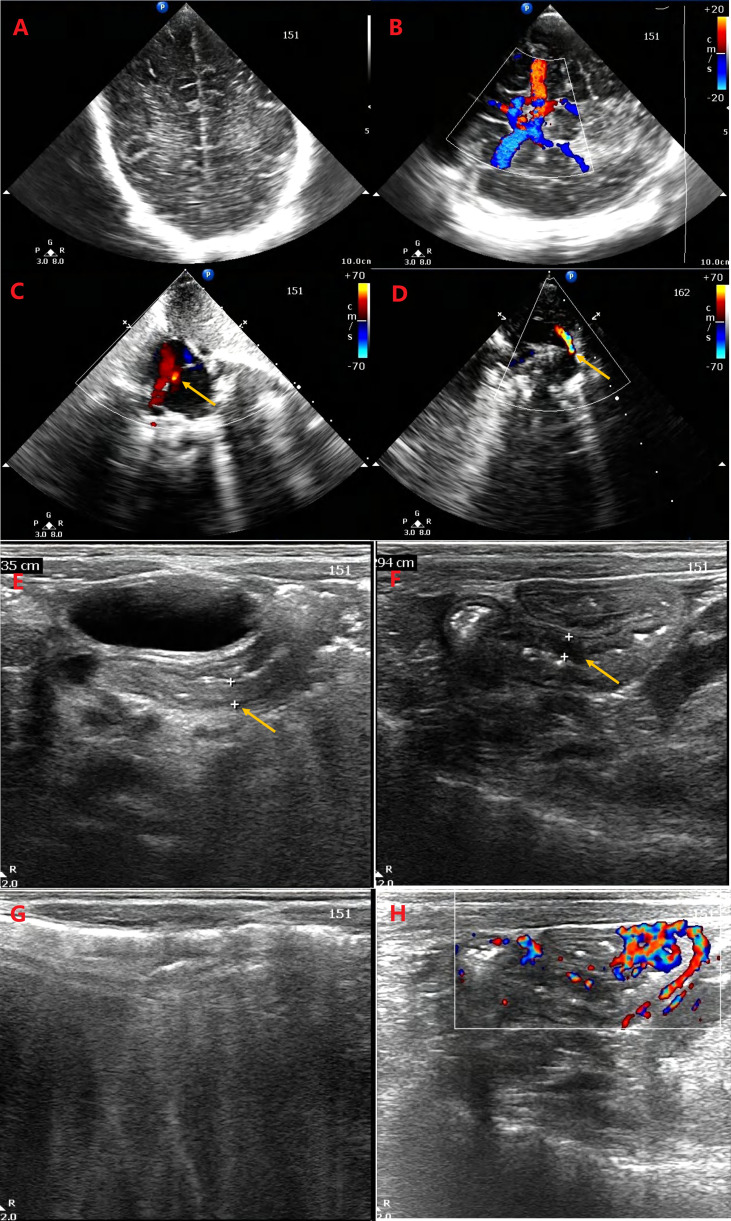
Ultrasonic Doppler resolution is applied for detecting the abdomen, heart, and brain. No obvious abnormality was detected on the brain sonogram **(A, B)**. The patent ductus arteriosus **(D)** and patent foramen ovale **(C)** were found. There are partial bowel flatulence of epigastrium, partial intestinal thickening of colon (0.335 cm, 0.294 cm) **(E, F)**, and a little of seroperitoneum. Intestinal gassiness **(G)** and blood flow in thickened intestinal wall **(H)** were detected.

With the increase in ultrasonic Doppler resolution, the technology is being applied to an increasing number of neonatal disease screenings due to its non-invasive, repeatable, and convenient nature. Therefore, the abdomen, heart, and brain were detected, and patent ductus arteriosus (PDA) and patent foramen ovale (PFO) were found; however, no obvious abnormalities were detected on the brain sonogram. In addition, partial bowel flatulence of the epigastrium, partial intestinal thickening of the colon (0.3 cm), and a small amount of seroperitoneum were observed (**Figure 2**).

### Treatment

When the newborn was hospitalized, fluid infusion, smectite powder, probiotics, and antibiotics (Mezlocillin and ceftriaxone) were applied for the diarrhea and infection. During the treatment course, the neonate had a fever with white cell count of up to 45.04*10^9^/L and, through blood and sputum culture, was detected to have *Staphylococcus epidermidis* and *Enterococcus faecium* infection, respectively. Therefore, vancomycin, meropenem, and immunoglobulin were ordered for the infection. When diarrhea was severe, fasting and parenteral nutrition were applied for the treatment. After the infection was under control, methylprednisolone (small dose) and budesonide (sustained-release capsules) were administered for anti-inflammatory effects, while infliximab and cyclosporine were prescribed for immunosuppressive balance. The patient is currently in a stable condition, with mild diarrhea and no infection. The entire pathogenesis and treatment process is summarized in **Supplementary Figure 1**.

## Material and methods

### Compliance with ethical standards

This study was approved by the Medical Ethics Committee of the Children’s Hospital affiliated with Shandong University (Ethics Approval No. ETYY-2014012). Clinical and laboratory examinations were performed on the probands after obtaining the written informed consent from their parents or guardians. All procedures in this study were performed in accordance with the Declaration of Helsinki.

### Next-generation sequencing and variant calling

Blood samples of the proband and her parents were collected in an EDTA vacutainer for DNA extraction using a QIAamp DNA Blood Midi Kit (Qiagen, Shanghai, China) and determined using a NanoDrop 2000 ultraviolet spectrophotometer (Thermo Fisher, USA). Using a NovaSeq 6000 platform (Illumina, United States), clinical exome sequencing with a GenCap MedE006 capture kit (MyGenostics, Beijing, China) was used to screen for mutations in the probands. The obtained mean exome coverage of the target regions was greater than 95% (>10 coverage; mean depth of over 100X). The sequencing data were aligned using Burrows–Wheeler Aligner software (BWA Version:0.7.10) with version GRCh37/hg19 of the human genome. SAM files were then converted to BAM. GATK’s RealignerTargetcreator and IndelRealigner were applied to local realignment, GATK’s BaseRecalibratorbase was applied to quality score recalibration, and variants were jointly called using GATK’s HaplotypeCaller in the “GENOTYPE_GIVEN_ALLELES” mode. Then, SNPs and indels were filtered using GATK’s VariantFiltration, and ANNOVAR was used to annotate the variants. To remove common variants (sub-allelic frequency >5%), variant frequencies were determined in 1000 genomes, ExAC, gnomAD, ESP6500, and in-house databases. Likely pathogenic variants associated with the patient-standardized HPO phenotype were prioritized for this study. The pathogenicity of novel variants was evaluated using SIFT, PolyPhen-2, MutationTaster, SpliceAI, and REVEL. Moreover, the variations reported in HGMD (http://www.hgmd.cf.ac.uk) and ClinVar (http://www.ncbi.nlm.nih.gov/clinvar) will be analyzed further. The variants identified in this study were classified according to the 2015 American College of Medical Genetics and Genomics (ACMG) guidelines (23–27). The suspicious variations were selected, and their segregation analysis was performed by Sanger sequencing. The function of the variants and their correlation with the disease phenotype were done by OMIM database, dbSNP database, Decipher database, and the literature. Schematic presentation of the detailed and comprehensive data interpretation process is shown in **Supplementary Figure 2**.

### Sanger sequencing to verify the mutations

The likely pathogenic variants identified by clinical exome sequencing in the probands using specific primers were validated by Sanger sequencing. Sanger validation primer sets were designed using Primer Premier v5.0 software. PCR amplification was performed using the AmpliTaq Gold 360 DNA polymerase (Applied Biosystems). The PCR products were further purified and sequenced using an ABI Prism 3700 automated sequencer (Applied Biosystems, Foster City, CA, USA).

### Bioinformatics analysis

Conservation analyses among multiple diverse species were performed using ClustalX software. Modeling of wild-type and mutant proteins was performed using the online SWISS-MODEL tool (http://www.swissmodel.expasy.org). Splicing patterns of putative splicing variants were predicted using the online RNA Splicer tool (https://rddc.tsinghua-gd.org/search-middle?to=SplitToolModel).

### RNA and cDNA extraction, Sanger sequencing

The total RNA extracted from the peripheral blood of the proband was stored in a TRIzol reagent (Invitrogen, USA) according to the manufacturer’s protocol. First, peripheral blood cells were lysed by vortexing in a TRIzol Reagent, a monophasic solution of phenol, guanidine isothiocyanate, and other proprietary components. The TRIzol Reagent maintains the integrity of the RNA because of the highly effective inhibition of RNase activity while disrupting cells and dissolving cell components during sample homogenization. Next, chloroform was added to separate the solution into aqueous and organic phases. RNA remained in the aqueous phase and was recovered by precipitation with isopropanol. The resulting nucleic acids were quantified using a NanoDrop 2000 ultraviolet spectrophotometer (Thermo Fisher Scientific, USA). cDNA was obtained from RNA reverse transcription using a Takara PrimeScript™ RT reagent Kit (TaKaRa) following the manufacturer’s instructions. The sequenced fragments, including the mutation c.3453C>T (p.Gly1151=) of the *PI4KA* gene, were amplified from the cDNA of the proband using a forward primer *PI4KA*-F (5’-TGCAGTCGCGGACAAAGTATTC-3’) and a reverse primer *PI4KA*-R (5’-GCTCCGGGTGTCCTGATTATC-3’). Later, the possible cDNA alterations caused by the c.3453C>T mutation were detected by Sanger sequencing, as described above.

### Minigene splicing assay

**Figure 3:**
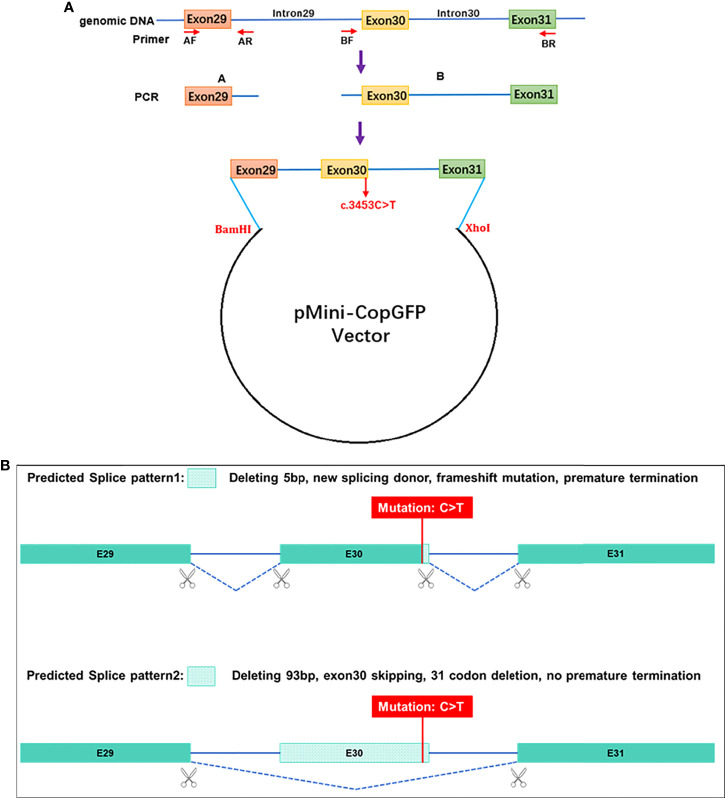
Schematic diagram for the synonymous mutation c.3453C>T affecting splicing. **(A)** Plasmid construction flow chart; **(B)** the online RNA Splicer tool predicted two splice patterns, pattern 1 with 5 bp deleting and pattern 2 with exon 30 (93 bp) skipping.

To verify the probable splicing effects caused by the c. 3453C > T mutation, a minigene splicing assay was performed *in vitro*. The minigene regions of the *PI4KA* gene spanning exon 29, intron 29, exon 30, intron 30, and exon 31 (**Figure 3A**) were amplified from the gDNA of the control using a forward primer (5′- AAGCTTGGTACCGAGCTCGGATCCGAATATCTGAACAAACATCAGAACTGGG -3′) with the restriction site BamHI and a reverse primer (5′- TTAAACGGGCCCTCTAGACTCGAGCTTTACTGCTAATGAGCATTGCGGTCAG -3′) with the restriction site XhoI. The amplified products were cloned into the pMini-CopGFP vector (Beijing Hitrobio Biotechnology Co., Ltd.) using a ClonExpress II One Step Cloning Kit (Vazyme, Nanjing, China). The cloned wild-type plasmid was validated using Sanger sequencing. Mutant plasmids were created by recombining the mutant fragments obtained with the mutagenesis primers *PI4KA*-MT-F (5′- GGtGAGGTGCTTCTCTTGTCCTTGCGCCCTAG-3′) and *PI4KA*-MT-R (5′- AAGAGAAGCACCTCaCCCGCGTAGCGGTTGCGC-3′). The mutant plasmid was validated using Sanger sequencing. The selected recombinant plasmids were transiently transfected into HEK293T cells using Lipofectamine 3000 (Invitrogen) following the manufacturer’s instructions. Total RNA was extracted from cells cultured for 48 h using a TRIzol reagent (Invitrogen, USA). RT-PCR was conducted using the MiniRT-F primer pair (5′- GGCTAACTAGAGAACCCACTGCTTA-3′) and MiniRT-R (5′-CTTTACTGCTAATGAGCATTGCG-3′). We analyzed the amplified PCR fragments by agarose gel electrophoresis and determined the isoforms by Sanger sequencing.

### Enzymatic activity measurement

Because *PI4KA* is a high-molecular-weight protein, plasmid construction and expression are very difficult. Therefore, wild-type or mutant-type human *PI4KA* (c.4878-6306) was constructed with the pcDNA3.1-C-Flag plasmid that contains the whole catalytic region and the partial Pleckstrin homology domain. The kinase catalytic site with the mutation p.Asp1957Ala was used as a positive control. HEK293T cells grown in 10-cm culture dishes were transfected with either the wild-type or mutant-type *PI4KA* plasmid using a transfection kit (Lipofectamine 3000, Invitrogen). Subsequently, immunoprecipitates from cell lysates with an anti-flag antibody (MA9019, Abmart) and Protein A/G PLUS-Agarose (A0421, Santa Cruz Biotechnology) were implemented. After acquiring the protein, the kinase activity was measured on the beads using 0.3 m ATP and 0.8 mM PI as substrates according to the protocol (ADP-Glo Kinase Assay, Promega). Western blotting was applied for detecting the expressed protein. Equal amounts of cell lysates (input) and the eluates of the beads were loaded on 10% SDS-PAGE gels and separated by gel electrophoresis. The first antibody was polyclonal rabbit Flag antibody (TT0053, Abmart), and the secondary antibody was Goat anti-rabbit IgG (A00098, GenScript). ECL Chemiluminescence (36208ES60, Yeasen) was applied for detecting the band imaging (ChemiScope 5300, Qinxiang, China).

## Results

### Genetic analysis

**Figure 4:**
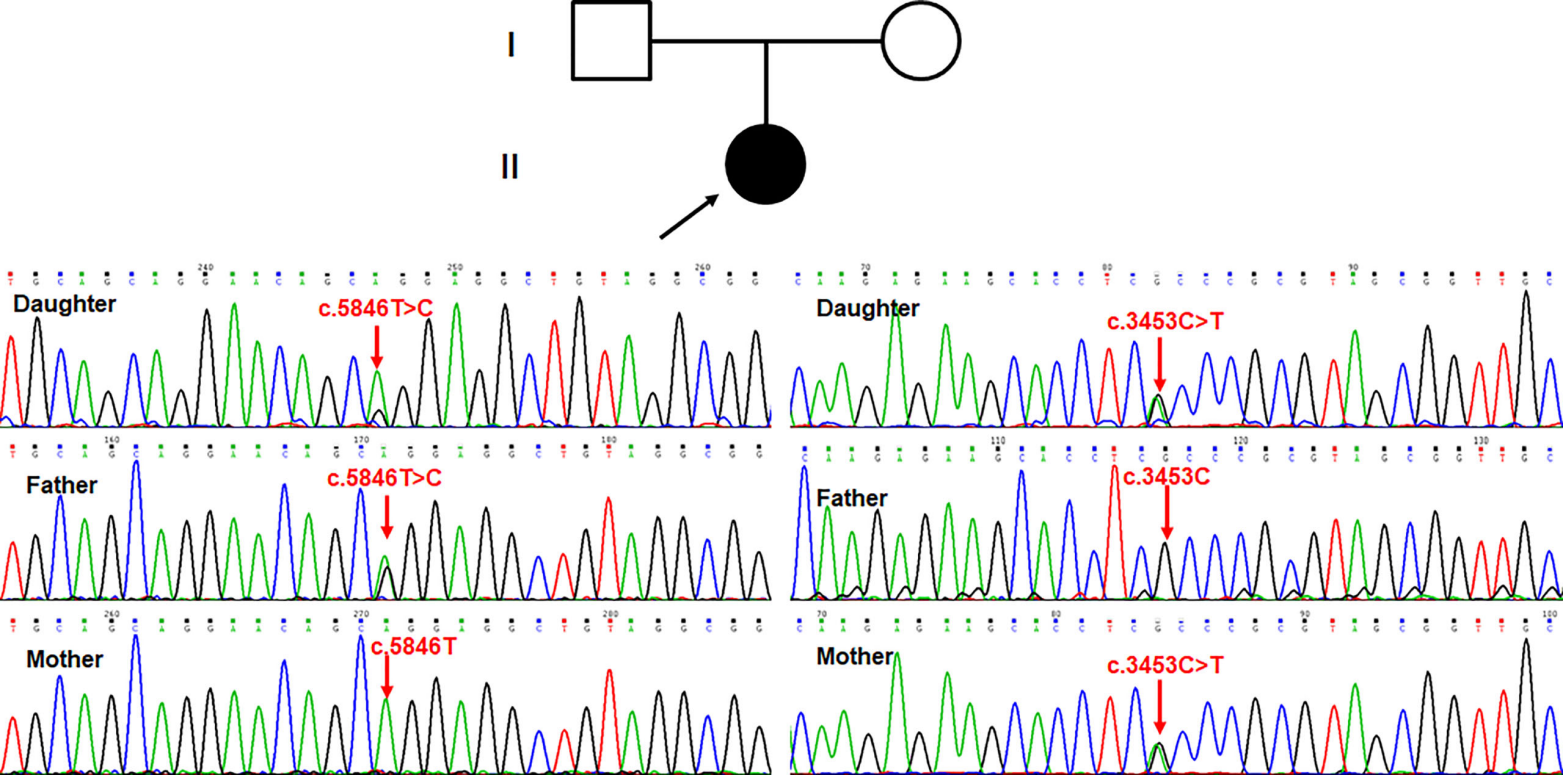
Sanger sequencing showed that c.5846T>C (p.Leu1949Pro) was paternal variant, while c.3453C>T (p.Gly1151=) was maternal variant.

According to the autosomal recessive models and clinical phenotypes of immunodeficiency disorders, compound heterozygous variants (c.5846T>C and c.3453C>T, NM_058004) detected by clinical exome sequencing were suspected to be disease-associated variants in the proband in the *PI4KA* gene, a known gene that can cause gastrointestinal defects and immunodeficiency syndrome 2 (OMIM: 619708) (**Supplementary Table 2**). The missense variant c.5846T>C (p.Leu1949Pro) was not reported in 1000 genomes, ExAC, Exome Variant Server, in-house databases, or published literature databases. *PI4KA* c.3453C>T (p.Gly1151=), a synonymous mutation, was absent from the 1000 Genomes Project, ExAC, ESC6500, and in-house database but has a minimal frequency in the global population of gnomAD (0.000003184). The NGS data have been deposited in NODE Bioproject (OEP003521). Sanger sequencing (**Figure 4**) showed that c.5846T>C (p.Leu1949Pro) was a paternal variant, while c.3453C>T (p.Gly1151=) was a maternal variant.

### Bioinformatics analysis

**Figure 5:**
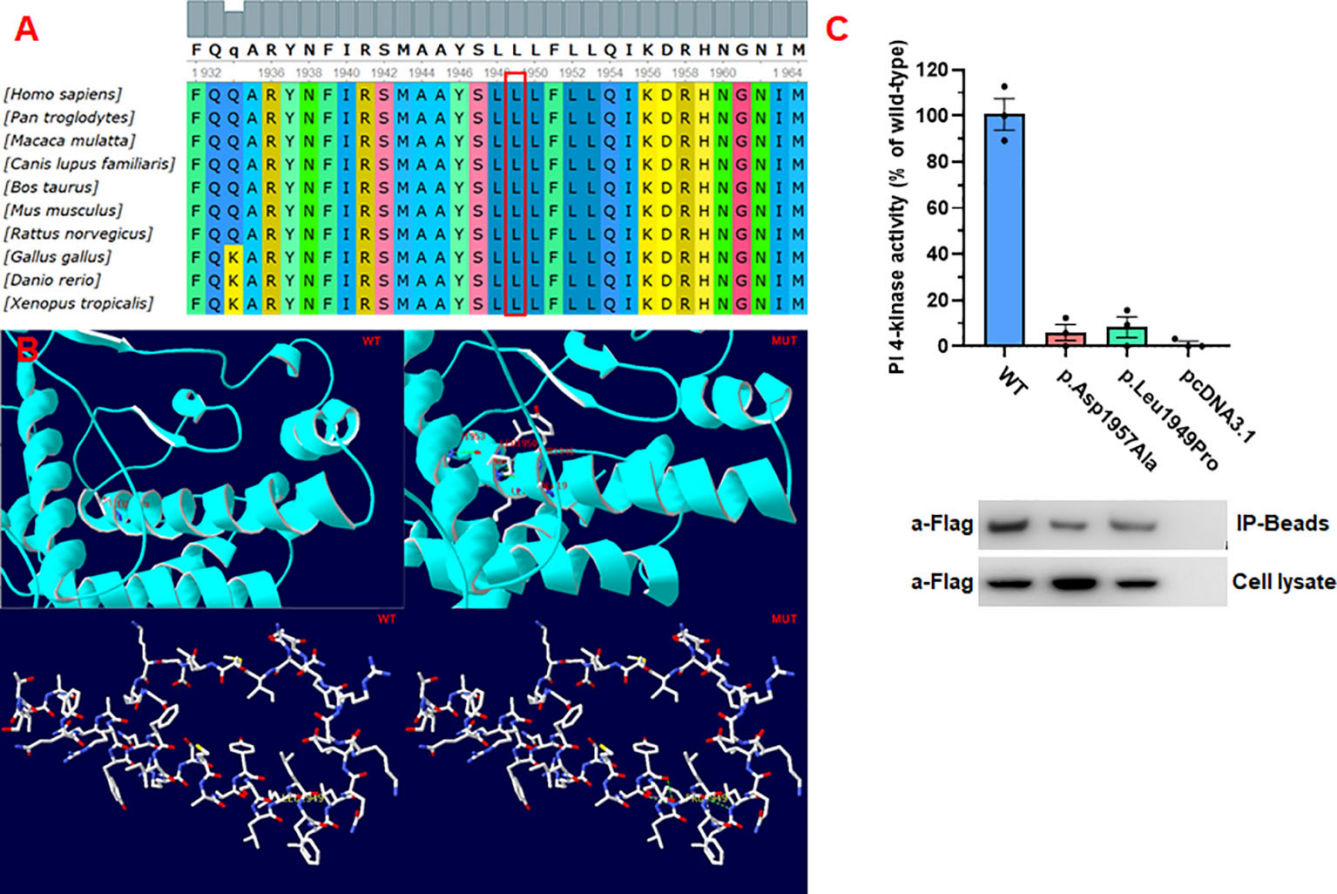
Pathogenicity of the missense mutations of *PI4KA*. **(A)** *In silico* analysis of p.Leu1949Pro in *PI4KA* shows the site p.Leu1949 highly conservative in different species. **(B)** 3D structure of wild type and mutant type of p.Leu1949Pro in *PI4KA*, and the mutation of p.Leu1949Pro changes the side strand structure and H-bond. **(C)** Functional studies of *PI4KA* c.5846T>C (p.Leu1949Pro) kinase activity.

Conservative analysis revealed that leucine at site 1949 (p.Leu1949) was highly conserved in multiple species (**Figure 5A**). Structural modeling showed changes in the side-strand structure and H-bond at residue 1949, in which leucine was substituted by proline (**Figure 5B**). The novel c.5846T>C (p.Leu1949Pro) variant of *PI4KA* was predicted to be deleterious by MutationTaster (0.999, disease-causing), REVEL, SIFT (0.005, damaging), and PolyPhen-2 (0.971, probably damaging). Since the synonymous variant c.3453C>T (p.Gly1151=) was located near the exon–intron junction, the SpliceAI and online RNA Splicer tools were used to assess the potential impacts on the splicing of this variant. The delta score of the c.3453C>T variant predicted by the deep learning algorithm SpliceAI was 0.98. Two possible abnormal splice patterns were predicted using the online RNA splicer tool, namely splice pattern 1 with 5 bp deletion and splice pattern 2 with exon 30 skipping, which could cause 31 codons missing without premature termination due to exon 30 containing 93 bp (**Figure 3B**).

### Splicing study of *PI4KA* c.3453C>T by RT-PCR Sanger sequencing of the proband

**Figure 6:**
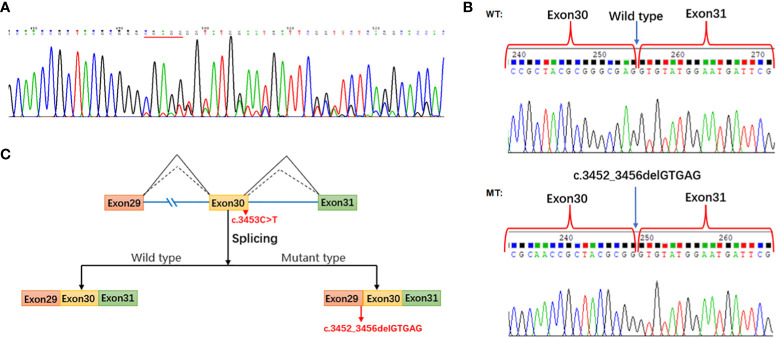
Splicing study of *PI4KA* c.3453C>T. **(A)** Sanger sequencing of RT-PCR for the proband peripheral blood mononuclear cells. **(B)** Splicing study by minigene assay; the wild-type fragment is 441 bp and the mutation type 436 bp; Sanger sequencing of RT-PCR for the plasmid expression. **(C)** Schematic of splicing for *PI4KA* c.3453C>T. WT, wild type; MT, mutation type.

First, we found that *PI4KA* mRNA is highly expressed in peripheral blood mononuclear cells (PBMCs) using the Human Protein Atlas (https://www.proteinatlas.org/), an online tool. The splicing result caused by *PI4KA* c.3453C>T was confirmed by RT-PCR Sanger sequencing of PBMC-derived RNA of the proband. The results revealed the deletion of five bases (GTGAG) in the *PI4KA* cDNA with the c.3453C>T variant (**Figure 6A**). However, the quantification of stranded DNA is only up to about a quarter of that of normal stranded DNA.

### Splicing study of *PI4KA* c.3453C>T by minigene assay

We conducted a minigene analysis of the wild and mutant types carrying *PI4KA* c.3453C>T to further characterize the abnormal splicing. Agarose gel electrophoresis of RT-PCR products showed a single band from the wild type (expected 441 bp) and mutant type (expected 436 bp) (**Supplementary Figure 3**). Sanger sequencing revealed a normal splicing isoform for the wild type and abnormal splicing for the mutant type (**Figure 6B**), consistent with splice pattern 1 (5 bp deletion, **Figure 3B**) predicted by the online RNA Splicer tool. Minigene analysis suggested that the c.3453C>T substitution can generate a new acceptor splice site (GT) in intron 30 of the *PI4KA* gene and lead to the deletion of five bases (GTGAG) in the *PI4KA* cDNA with the c.3453C>T variant, predicted to result in a truncated protein (p.Gly1151GlyfsTer17) (**Figure 6C**).

### Functional studies of *PI4KA* c.5846T>C (p.Leu1949Pro) kinase activity

To determine the activity of the mutant enzyme (p.Leu1949Pro), we generated the p. Leu1949Pro mutant form of the human *PI4KA* enzyme and compared its activity with that of the wild-type enzyme (positive control) and the negative control (p.Asp1957Ala). As shown in **Figure 5C**, the mutant enzyme (p.Leu1949Pro) showed a small or no measurable catalytic activity.

## Discussion

Previous studies have shown that *PI4KA* plays a key role in hepatitis C virus (HCV) infection; the major mechanism is *PI4KA* and the metabolite PI-4-P can support HCV replication in host cells (28, 29). Moreover, the *PI4KA* polymorphism, *PI4KA*-rs165854, has the potential to jointly modulate schizophrenia patients with poor antipsychotic response (30). In addition, PI4KIIIα protein enrichment has mediated the synaptic and gene regulatory mechanisms in 22q11.2 copy number variants (CNVs)-related diseases. The 22q11.2 deletion syndrome is a high risk for schizophrenia, and region reciprocal duplication results in autism spectrum disorder (ASD) and schizophrenia (31, 32). *PI4KA* expression was influenced by diet *in vivo* and was downregulated through a high fatty acid (FA) composition diet (33). Although the PI4KIIIα protein has a wide range of effects, its pathogenicity has not been reported. With recent advancements in sequencing technology, it has finally been demonstrated that *PI4KA* gene mutation can cause single-gene recessive genetic diseases (8–10).

The protein EFR3, also known as homolog A/B (EFR3A/B), which can be palmitoylated and plays a catalytic role, recruits the PI4KIIIα complex PI4KIIIα-FAM126-TTC7 to the plasma membrane (34) (**Figure 1B**). Except for the catalytic subunit in the PI4KIIIa-FAM126-TTC7-EFR3 molecular complex, all other subunits were encoded by two different genes (A/B). Different subunits are expressed in different tissues, and it has been demonstrated that TTC7A is highly expressed in the small intestine and colon, whereas TTC7B is mainly expressed in the brain (9). The reported p.Tyr1623Asp alteration in PI4KIIIa more strongly affects the stability of TTC7A than TTC7B (9). The above results can prompt us to deliberate the intrinsic mechanism by which variances in the diverse domains of PI4KIIIα can cause different diseases in humans (OMIM: 616531, 619621, and 619708). Pagnamenta et al. (10) reported three affected fetuses, conceived by unrelated parents of European descent, with multiple congenital abnormalities, including anomalies perisylvian polymicrogyria, cerebellar hypoplasia, and arthrogryposis, and identified compound heterozygous mutations p. Arg796Ter and p.Asp1854Asn of the *PI4KA* gene from the father and the mother in aborted fetal samples, respectively. The high levels of PI lipids and PI4Ks in the brain suggest their importance in brain development (35). The role of the PI3K-AKT-mTOR signaling pathway in brain development is well orchestrated, and PI4KIIIα is an upstream partner of PI3Ks in the signaling cascade (36). Hereafter, Verdura et al. (8) (2021) identified a neurodevelopmental disorder caused by homozygous or compound heterozygous mutations in the *PI4KA* gene in 10 unrelated patients, among whom patients 1–8 had shown the anomalies perisylvian polymicrogyria, cerebellar hypoplasia, and arthrogryposis (OMIM: 616531); patients 9 and 10 with compound heterozygous mutations in the *PI4KA* gene had autosomal recessive spastic paraplegia-84 (OMIM: 619621), which suggests that PI4KIIIα and phosphoinositide signaling played major roles in myelination and brain development. Simultaneously, Salter et al. (2021) found that the homozygous gene mutation c.4867T>G (p.Tyr1623Asp) of *PI4KA* caused GIDID2 in a large multigenerational consanguineous Amish kindred with five infants affected (9). GIDID is characterized by intestinal atresia and immunodeficiency, and thus two subtypes have been identified until now—GIDID1 (OMIM: 243150) and GIDID2—caused by mutations in the *TTC7A* and *PI4KA* genes, respectively, surprisingly concerning in which the GIDID-related two genes were on the kinase PI4KIIIa-TTC7-FAM126-EFR3 complex. This complex plays an important role in normal intestinal and immune cell development. Meanwhile, disruption of the gene function in the phosphatidylinositol metabolism pathway can cause different gastrointestinal phenotypes in animal models, such as *CDIPT*, *PIK3C3*, *PI3K*, *PI4KA*, and *TTC7A* (9, 37–39).

We reviewed all reported gene variances and clinical characteristics of PI4KIIIα resulting in different genetic disease phenotypes, and every domain of PI4KIIIα contained the variances, especially in the catalytic domain (**Figure 1C**; **Supplementary Table 1**). Twenty missense mutations and 10 truncating mutations have been reported so far. Our study discovered that the variance c.3453C>T (p.Gly1151=) is a synonymous mutation; however, it can legitimately cause mRNA alternative splicing, including 5 bp deletion in the mRNA, and lead to a frameshift and premature translation termination. Simultaneously, the amino acid residue p.Leu1949 in the catalytic domain is highly conserved among species, and the missense variance c.5846T>C (p.Leu1949Pro) can result in complete loss of function in the kinase activity assay *in vitro* (**Figure 4**). However, *in vitro* functional experiments may not completely show protein dysfunction; the enzyme activity with the p.Asp1854Asn variance located in the PI4KIIIα catalytic domain was almost undetectable in Pagnamenta’s report (10). However, Verdura et al. (8) found that a consanguineous marriage family gave birth to a boy with the homozygous mutation c.5560G>A (p.Asp1854Asn) and represented a milder phenotype, suggesting that p.Asp1854Asn could not cause the complete loss of function and possibly the remaining catalytic activity was not enough to sustain life for the aborted fetus. Meanwhile, the boy with the homozygous mutation c.5560G>A (p.Asp1854Asn) had polymicrogyria, similar to the fetuses, suggesting that the Asp1854Asn variant may be specifically associated with brain abnormalities. In our study, the protein with the substitution p.Leu1949Pro showed no catalytic activity *in vitro*; however, whether any remaining activity occurs *in vivo* needs further validation.

Synonymous mutations are often considered non-pathogenic because they do not alter the amino acid of the encoded protein; however, synonymous mutations at non-canonical splicing sites can lead to abnormal splicing, and reports about the relationship between synonymous mutations and abnormal splicing have increased recently (40). In our study, we found that the child has the synonymous variance c.3453C>T (p.Gly1151=) from the mother, and the location is near the splicing site at the 5’ end of the intron starts with the GT called donor, and the mutation creates a new donor site. Regarding the cDNA level with c.3453C>T only up to 1/4 of the normal cDNA strand, there was probably nonsense-mediated mRNA decay (NMD) or other splicing changes for the synonymous variance. Therefore, we confirmed the mRNA splicing *in vitro* using the minigene method. NMD is an mRNA quality-control mechanism in all eukaryotes that can survey newly synthesized mRNA and degrade premature termination codons (PTC). It is well known that the PTC can produce truncated proteins that can be detrimental and cause disease, and NMD is estimated to downregulate about one-third of disease-causing mRNAs. Our experiment on detecting mRNA in the patient’s blood also verified the role of NMD in maintaining cellular homeostasis. Otherwise, PI4KIIIa is necessary for HCV infection and replication (41). The database shows that the synonymous c.3453C>T mutation has a certain mutation frequency (0.0318‰) in the normal population. Whether the normal population with low expression of PI4KIIIa could resist HCV infection needs to be further studied.

Considering the functional loss of PI4KIIIα giving rise to three different syndromes, our propositus showed immunodeficiency-associated symptoms of neonatal sepsis, diarrhea, fever, decreased immunoglobulin, and B lymphopenia. Perhaps, other motions and neurological abnormalities will present with increasing age. Fortunately, the patient has been improving with age and treatment, and other syndromes have disappeared except for diarrhea. To date, TTC7A- and PI4KIIIα-related GIDID1 and GIDID2 are untreatable disorders in which surgical intervention could improve intestinal atresia without affecting the immunodeficiency outcome. Leflunomide is a disease-modifying anti-rheumatic drug (DMARD) used to treat patients with inflammatory conditions. It can reduce apoptosis and activate AKT signaling in *TTC7A*-KO cells, restore gut motility, reduce intestinal tract narrowing, and increase intestinal cell survival in ttc7a^-/-^ zebrafish, all of which are repurposed with leflunomide for the treatment of TTC7A deficiency (42). As TTC7A is known to interact with PI4KIIIa, leflunomide might also be effective in patients with PI4KIIIa deficiency suffering from intestinal disorders. Drug development for TTC7 and FAM126 in the kinase PI4KIIIa-TTC7-FAM126-EFR3 complex can be a strategy for treating PI4KIIIa deficiency.

In conclusion, according to the clinical characteristics and gene mutations with functional verification, pediatricians diagnosed children with combined immunodeficiency and intestinal disorders close to GIDID2 (OMIM: 619708). Medicine such as immunomodulators was prescribed to balance immune dysregulation. However, leflunomide was not prescribed because there was no evidence of its application. This study is the first report of a synonymous mutation in the *PI4KA* gene that influences alternative splicing. Our findings expand the mutation spectrum leading to PI4KIIIa deficiency-related diseases and provide exact information for genetic counseling.

## Data availability statement

The data presented in the study are deposited in the NODE Bioproject repository, accession number OEP003521. The direct link is https://www.biosino.org/node/project/detail/OEP003521.

## Ethics statement

The studies involving human participants were reviewed and approved by ETYY-2014012. Written informed consent to participate in this study was provided by the participants’ legal guardian/next of kin. Written informed consent was obtained from the minor(s)’ legal guardian/next of kin for the publication of any potentially identifiable images or data included in this article.

## Author contributions

KZ, DW, ZG, and XL designed and wrote the manuscript. KZ, LK, DC, and HY collected the clinical data. DW, HZ, LB, and XZ constructed the plasmid and performed the experiments. YLv, MG, and YL executed NGS. HP and QL collected the images. All authors contributed to the article and approved the submitted version.

## Funding

This work was financially supported by the Natural Science Joint Foundation of Shandong Province (ZR2021LSW010) and Horizontal Project of Shandong University (3450020001). The authors are grateful to the patients and their parents for their contribution to this study.

## Acknowledgments

The authors would like to thank all the family members for their participation and cooperation in this study. The authors also thank Yinggang Liu for assistance in data collection.

## Conflict of interest

The authors declare that the research was conducted in the absence of any commercial or financial relationships that could be construed as a potential conflict of interest.

## Publisher’s note

All claims expressed in this article are solely those of the authors and do not necessarily represent those of their affiliated organizations, or those of the publisher, the editors and the reviewers. Any product that may be evaluated in this article, or claim that may be made by its manufacturer, is not guaranteed or endorsed by the publisher.

## Supplementary material

Supplementary Figure 1 The entire pathogenesis and treatment process.

Supplementary Figure 2 Data interpretation pipeline for whole exome sequencing.

Supplementary Figure 3 Splicing study by minigene assay. The agarose gel electrophoresis was applied for detecting the wild-type fragment 441 bp and the mutation type 436 bp.
