# Supplementary figures and images for: A synonymous mutation in PI4KA impacts the transcription and translation process of gene expression
Source: Front Immunol. 2022 Oct 19;13:987666. doi: 10.3389/fimmu.2022.987666 (PMC9627211; doi:10.3389/fimmu.2022.987666)

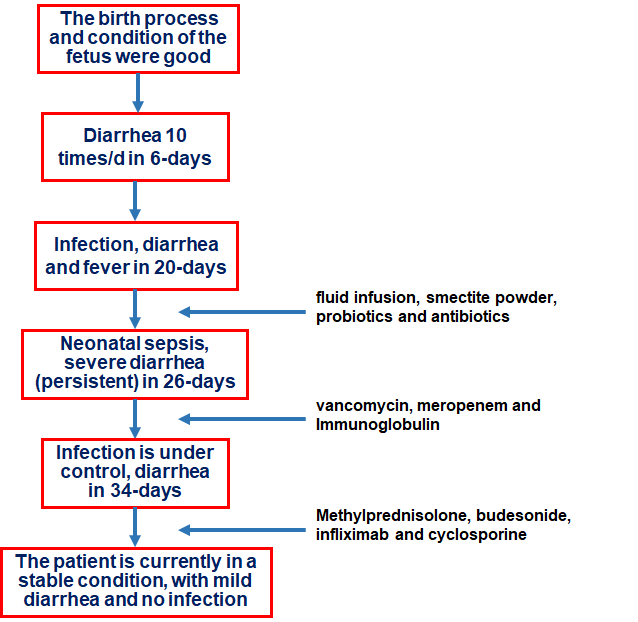

Supplement: Supplementary Figure 1 — The entire pathogenesis and treatment process. [file Image_1.tif]

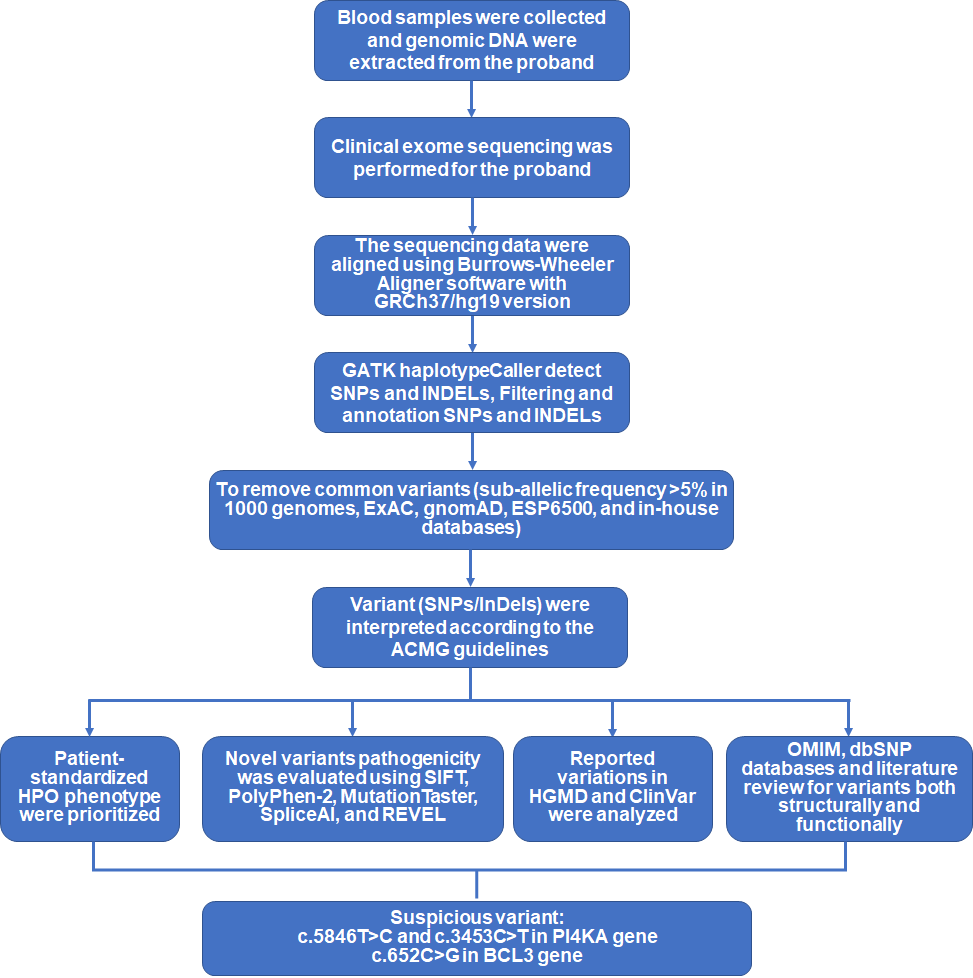

Supplement: Supplementary Figure 2 — Data interpretation pipeline for whole exome sequencing. [file Image_2.tif]

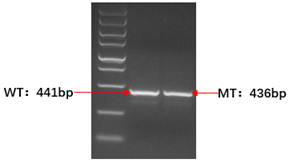

Supplement: Supplementary Figure 3 — Splicing study by minigene assay. The agarose gel electrophoresis was applied for detecting the wild-type fragment 441 bp and the mutation type 436 bp. [file Image_3.tif]
