# Supplementary material for: A synonymous mutation in PI4KA impacts the transcription and translation process of gene expression
Source: Front Immunol. 2022 Oct 19;13:987666. doi: 10.3389/fimmu.2022.987666 (PMC9627211; doi:10.3389/fimmu.2022.987666)
Supplement: Supplementary file 4 [file Table_1.docx]

| **Supplementary Table 1 The clinical and laboratory features of the reported patients with biallelic *PI4KA* variants** | | | | | |
| --- | --- | --- | --- | --- | --- |
| **General information** | **Family 1^[1]^** | **Family 2^[1]^** | **Family 3^[1]^** | **Family 4^[1]^** | **Family 5^[1]^** |
| Gender | Female | Male | Female | Female | Male |
| Ethnicity | Caucasian | Caucasian | Caucasian | Caucasian | Caucasian |
| Age at onset | Newborn | Newborn | 6months | 1year | Newborn |
| Age at exam | 4years | 3years | 13years | 19years | 10years |
| **Gene mutations** |  |  |  |  |  |
| Variant cDNA | c.2624dupC  c.3454G>A | c.5773G>C  c.5773G>C | c.3884A>G  c.6122T>C | c.3592G>A  c.6156_6159delGACA | c.1852C>T  c.4990G>A |
| Protein | p.Pro876SerfsTer36  p.Glu1152Ly | p.Gly1925Arg  p.Gly1925Arg | p.His1295Arg  p.Met2041Thr | p.Ala1198Thr  p.Thr2053SerfsTer4 | p.Arg618Ter  p.Asp1664Asn |
| **Neurological symptoms** |  |  |  |  |  |
| Motor signs | Spastic tetraparesis | Hypotonia | Spastic paraparesis | Spastic tetraparesis | Spastic paraparesis |
| Ataxia | + | - | + | + | + |
| Epilepsy | ++(IS) | ++ | + | + | - |
| Additional signs | Dystonia, choreoathetosis, hyperexcitability, nystagmus | Axonal sensory neuropathy, nystagmus, startle | Irritability (first months), tremor, very smiley, nystagmus | Tremor, nystagmus |  |
| **Development** |  |  |  |  |  |
| Motor and Walking | Severely delayed and Not achieved | Severely delayed and Not achieved | Severely delayed and Not achieved | Delayed and Delayed | Delayed and 2.8 years |
| Language | None | None | Delayed | Single words | Delayed |
| Intellectual disability | Severe | Severe | Severe | Mild | Mild |
| **Neuroimaging** | Diffuse hypomyelination, WM atrophy, ventriculomegaly, thin CC, brainstem and cerebellar hypoplasia | Diffuse hypomyelination, WM atrophy, ventriculomegaly, thin CC | Diffuse hypomyelination, dysplastic thin CC, cerebellar atrophy with calcifications | Diffuse hypomyelination, thin CC, cerebral, brainstem and cerebellar atrophy | Delayed myelination, dysplastic CC, cerebral atrophy, ventriculomegaly |
| **Gastrointestinal features** |  |  |  |  |  |
| Onset age | NA | NA | NA | NA | 9years |
| Multiple intestinal atresia | ✘ | ✘ | ✘ | ✘ | ✘ |
| Inflammatory bowel disease | ✘ | ✘ | ✘ | ✘ | ✘ |
| Other features | ✘ | ✘ | gastroesophageal reflux disease | ✘ | constipation |
| **Immunological features** | repetitive respiratory infections, otitis and pneumonia | recurrent bronchitis episodes and pneumococcal bacteremia | acute otitis media and pneumonia, hypogammaglobulinemia with a low CD19+ B-cell count | none | Impetigo, juvenile idiopathic arthritis |
| **Other clinical features** |  | bilateral cryptorchidism | atrial septal defect | none |  |
| **General information** | **Family 6^[1]^** | **Family 7^[1]^** | **Family 8^[1]^** | **Family 9^[1]^** | **Family 10^[1]^** |
| Gender | Male | Female | Male | Male | Male |
| Ethnicity | Asian/Caucasian | Caucasian | Turkish | Caucasian | Latin American |
| Age at onset | Newborn | 4months | Newborn | 17years | 2years |
| Age at exam | 6years | 11years | 5years | 40years | 18years |
| **Gene mutations** |  |  |  |  |  |
| Variant cDNA | c.5116+1G>A  c.5960A>G | c.1414A>C  c.355C>T | c.5560G>A  c.5560G>A | c.5459_5461delAAG  c.6156_6159delGACA | c.4666G>A  c.5159C>T |
| Protein | p.?  p.Asn1987Ser | p.Ser472Arg  p.Arg119Trp | p.Asp1854Asn  p.Asp1854Asn | p.Glu1820del  p.Thr2053SerfsTer4 | p.Val1556Met  p.Thr1720Ile |
| **Neurological symptoms** |  |  |  |  |  |
| Motor signs | Spastic tetraparesis | Spastic paraparesis | Spastic tetraparesis | Spastic paraparesis | Spastic paraparesis |
| Ataxia | + | + | - | - | - |
| Epilepsy | + | - | ++ | - | - |
| Additional signs | Hand flapping when excited | Stereotypic movements, ocular dyspraxia | Dystonia |  |  |
| **Development** |  |  |  |  |  |
| Motor and Walking | Delayed and 4.5 years | Delayed and 5years | Severely delayed and Not achieved | No delay and Normal | Delayed and 15 months |
| Language | None | Delayed | None | Normal | Normal |
| Intellectual disability | Severe | Severe | Severe | Normal | Mild |
| **Neuroimaging** | Delayed myelination, dysplastic CC, brainstem and cerebellar atrophy | Delayed myelination, external hydrocephalus, cerebellar atrophy | Bilateral perisylvian polymicrogyria | Arachnoid cyst of the posterior fossa, cervical spinal cord atrophy | Cervical spinal cord atrophy |
| **Gastrointestinal features** |  |  |  |  |  |
| Onset age | NA | NA | NA | 21years | NA |
| Multiple intestinal atresia | ✘ | ✘ | ✘ |  | ✘ |
| Inflammatory bowel disease | ✘ | ✘ | ✘ | Staged biopsies of the colon described fragments of the colonic mucosa with moderate inflammatory infiltrate and the presence of eosinophils in the lamina propria. | ✘ |
| Other features | ✘ | gastroesophageal reflux | Recurrent vomiting and gastroesophageal reflux | ✘ | ✘ |
| **Immunological features** | - | - | recurrent respiratory infections | ileocolonic Crohn’s disease with a stenosing-inflammatory pattern and a steroid-dependent course. | - |
| **Other clinical features** | Bilateral cryptorchidism, minor cardiac, renal malformations | not achieved urinary or faecal continence. | Bilateral cryptorchidism |  |  |
| **General information** | **Family 11(13 patients)^[2]^** | **Family 12(3 patients, 3 fetus)^[3]^** | **Family 13^[2]^** | **Family 14^[2]^** | **Family 15^[2]^** |
| Gender | 5Females+8males | 3Females+3abortion fetus | Female | Female | Female |
| Ethnicity | Amish | European | Turkish | Indian | German |
| Age at onset | Newborn | Antenatal | First day of life | 2 yrs | First week of life |
| Current age | Dead | Dead or Abortion | 13 years | 5 years | 24 years |
| **Gene mutations** |  |  |  |  |  |
| Variant cDNA | c.4867T>G  c.4867T>G | c.2386C>T  c.5560G>A | c.5560G>A  c.5560G>A | c.6065delG  c.5774G>A | c.5810A>G  c.5298_5299delTG |
| Protein | p.Tyr1623Asp  p.Tyr1623Asp | p.Arg796Ter  p.Asp1854Asn | p.Asp1854Asn  p.Asp1854Asn | p.Arg2022GlnfsTer36  p.Gly1925Glu | p.Tyr1937Cys  p.Glu1767fsTer14 |
| **Neurological symptoms** |  |  |  |  |  |
| Motor signs | Not known | Not known | Elevated spasticity for lower limbs | progressive spastic diplegia | progressive limb spasticity lower>upper |
| Ataxia | Not known | Not known | - | - | Ataxia with intention tremor and dysmetria |
| Epilepsy | Not known | Not known | Epileptic encephalopathy, West-syndrome during infancy. Tonic, myoclonic and atypical absence seizures | ✘ | Age 7 yrs: GTC, controlled with Sultiam; |
| Additional signs | Not known | Bilateral talipes equinovarus; | Optic atrophy, convergent strabismus, Hearing loss | Drinks or eats from a spoon. Unable to chew | Decreased visual acuity, nystagmus |
| **Development** |  |  |  |  |  |
| Motor and Walking | Not achieved | Not achieved | Rolling, unable to sit or stand, no head control. Truncal hypotonia, limb spasticity, Severe impairment of fine motor function, grasping movements not possible | Weight bears, not crawling, unable to maintain 4 point stance, Fine tremors that affects coordination |  |
| Language | Not achieved | Not achieved | No speech or vocalisations. Communicates via talker. Laughs and cries | First words ~18 months. Several words, understands simple commands. No dysarthria | Speaks several words, better receptive language skills |
| Intellectual disability |  |  | Severe | Mild | Moderate |
| **Neuroimaging** | Not known | Bilateral asymmetric perisylvian  Cerebellar hypoplasia, small pons Asymmetric cerebral ventriculomegaly | Immature gyral pattern for Polymicrogyria  Diffuse T2-elevation of white matter signal in the entire supratentorial white matter; Thin corpus callosum | Profound hypomyelination.  Myelin present in the posterior limbs of the internal capsules, lateral thalami, and within the corpus callosum and brainstem, but the rest of the brain appears hypo-myelinated. No progression in myelination seen in serial imaging with advancing age | Mild atrophy of pons and medulla oblongata, mild cerebellar atrophy; Diffuse T2-elevation of white matter signal in the entire supratentorial white matter with loss of parieto-occipital white matter. Severe thinning of the corpus callosum |
| **Gastrointestinal features** |  |  |  |  |  |
| Onset age | Newborn | Antenatal, gestation unknown | 3 yrs (anaemia) | 6 wks | 19 yrs |
| Multiple intestinal atresia | bowel obstruction including abdominal distension, bilious vomiting and failure to pass meconium. | ✘ | ✘ | ✘ | ✘ |
| Inflammatory bowel disease | ✔ | Not known | Persistent iron deficiency anaemia, raised faecal calprotectin | Colitis,  no small bowel involvement | Pancolitis with proctocolectomy and terminal ileum resection |
| Other features | - | - | - | - | - |
| **Immunological features** | X:28 with mild intermittent lymphopaenia, X:1 with severe immunodeficiency severe T-cell lymphopaenia, affecting CD8+ T cells more than CD4+ T cells, moderate B- and NK-cell lymphopaenia and agammaglobulinaemia | Not known | B cell lymphopenia, hypogammaglobulinaemia, moderate lymphopenia with marked reduced B- and NK cells. Mildly reduced activated and absent CD4+ T cells, Expansion of senescent CD57+ CD8+ T cells, Elevated transitional and CD21 low B cells as well as reduced naive B cells. Follicular non-Hodgkin lymphoma (non-paediatric) grade 3a | Autoimmune enteropathy | ✘ |
| **Other clinical features** | Not known | Abnormal development of kidneys and lungs | Chronic lung disease; interstitial lung emphysema and bronchiectasis,  scoliosis, hyperthyroidism,  poor sleep | Rectovaginal fistula | Kyphosis, delayed dentition, increased bleeding tendency, prolonged menstruation required blood transfusions, Goldnetz™-endometrial ablation performed aged 24 yrs |
| **General information** | **Family 16^[2]^** | **Family 17^[2]^** | **Family 18^[2]^** | **The proband** |  |
| Gender | Male | Female | Female | **Female** |  |
| Ethnicity | Turkish | German | Italy | **Chinese** |  |
| Age at onset | 6 months | 6 months | 2 years | **6days** |  |
| Current age | 11 years | 21 years | 8 years | **3months** |  |
| **Gene mutations** |  |  |  |  |  |
| Variant cDNA | c.5197C>T  c.5197C>T | c.2330T>C  c.3571C>T | c.1696C>T  c.5423A>C | **c.3453C>T**  **c.5846T>C** |  |
| Protein | p.Arg1733Trp  p.Arg1733Trp | p.Leu777Pro  p.Gln1191Ter | p.Arg566Ter  p.Lys1808Thr | **p.G1151=**  **p.Leu1949Pro** |  |
| **Neurological symptoms** |  |  |  |  |  |
| Motor signs | Elevated spasticity for lower limbs | Elevated spasticity for lower limbs | Elevated spasticity for lower limbs | **Not achieved** |  |
| Ataxia | Mild intention tremor and dysmetria, mild ataxia | Intention tremor and dysmetria | Hand tremor, axial hypotonia | **Not achieved** |  |
| Epilepsy | ✘ | Age 6 yrs: GTC, controlled with Sultiam. Relapse age 13 yrs: GTC and stimulus-induced myoclonus, controlled with Levetiracetam and Valproic acid | Age 3 yrs: recurrent GTC, controlled with Depakin. Seizure free by 8 yrs | **✘** |  |
| Additional signs | Normal vision, saccadic pursuit, fine pendular nystagmus and gaze-evoked nystagmus | Decreased visual acuity, pendular nystagmus | Mild horizontal nystagmus,  visual evoked potentials showed abnormal retinal-cortical conductions | **Not detected** |  |
| **Development** |  |  |  |  |  |
| Motor and Walking | Crawling and standing with support (age 15 months), walks with a retrowalker (6 yrs), scissoring of legs,  climbs onto sofa, Manipulates objects with difficulty | Sitting without support (age 5 yrs), walking with support (age 6 yrs), loss of walking with support (age 12 yrs), Manipulates objects with difficulty | Never walked independently, few steps with support (8 yrs), Manipulates objects with difficulty | **Not detected** |  |
| Language | Speak 2-3 word sentences, understands 2 languages.  Mild dysarthria | Speaks several words, better receptive language skills. Dysarthria | Mild language delay | **Not detected** |  |
| Intellectual disability | Moderate | Moderate | Mild |  |  |
| **Neuroimaging** | Diffuse T2-elevation of white matter signal in the entire supratentorial white matter with some loss of parieto-occipital white matter. Some T1-hypointense white matter areas. Slight thinning of the corpus callosum | Mild atrophy of pons and medulla oblongata, mild cerebellar atrophy; Diffuse T2-elevation of white matter signal in the entire supratentorial white matter with loss of parieto-occipital white matter; Some T1-hypointense white matter areas; Severe thinning of the corpus callosum; Progressive supratentorial atrophy | Diffuse T2 hyperintensity in the bilateral cortical regions; Mild corpus callosum hypoplasia and mild enlargement of subarachnoid spaces | **no obvious abnormalities were detected on the brain sonogram** |  |
| **Gastrointestinal features** |  |  |  |  |  |
| Onset age | NA | NA | NA | **6days** |  |
| Multiple intestinal atresia | ✘ | ✘ | ✘ | ✘ |  |
| Inflammatory bowel disease | ✘ | ✘ | ✘ | ✔ |  |
| Other features | - | - | - | **refractory diarrhea** |  |
| **Immunological features** | ✘ | ✘ | ✘ | **Hypogammaglobulinemia, B lymphopenia, T lymphocyte normal** |  |
| **Other clinical features** | Pronounced kyphosis | Pronounced kyphosis, Depressive mood, anxiety disorder, refusal to eat. | Left equinus foot deformity | **ductus arteriosus, patent foramen ovale** |  |

**Abbreviations:** wks, weeks; yrs, years; g, grams; NA, not available; ✔, indicates presence of a feature in an affected subject; ✘, indicates absence of a feature in an affected subject; GTC, Generalised tonic clonic seizures. CC, corpus callosum.

Centiles calculated from UK-WHO data.

| **Supplementary Table 2: Variants identified from clinical-exome sequencing** | | | | | | | | | |
| --- | --- | --- | --- | --- | --- | --- | --- | --- | --- |
| **Gene** | **GRChr37:g.** | **Transcript; exon** | **c. ( p.)** | **Zygosity/ inherited from** | **Pop Freq** | ***In silico* predictions** | **ACMG Pathogenicity** | **Disease/phenotype (inheritance patterns)** | **Reasons for excluding variant** |
| *PI4KA* | chr22:21065706 | NM_058004;exon51 | c.5846T>C (p.L1949P) | het/ paternal | - | D | Uncertain | 1.Gastrointestinal defects and immunodeficiency syndrome 2 (AR); 2.Polymicrogyria, perisylvian, with cerebellar hypoplasia and arthrogryposis (AR); 3.Spastic paraplegia 84, autosomal recessive (AR) | - |
| *PI4KA* | chr22:21098919 | NM_058004;exon30 | c.3453C>T (p.G1151=) | het/ maternal | 0.0000318 | - | Uncertain |  |  |
| *BCL3* | chr19:45260406 | NM_005178;exon4 | c.652C>G (p.P218A) | het/ maternal | - | B | Uncertain | Leukemia/lymphoma, B-cell, 3 (-) | Phenotype and inheritance mismatch |
| *PLEC* | chr8:144993491 | NM_000445;exon33 | c.10579C>T  (p.R3527C) | het/- | 0.0008 | D | Likely pathogenic | 1. ?Epidermolysis bullosa simplex 5D, generalized intermediate, autosomal recessive (AR); 2. Epidermolysis bullosa simplex 5A, Ogna type (AD); 3. Epidermolysis bullosa simplex 5B, with muscular dystrophy (AR); 4. Epidermolysis bullosa simplex 5C, with pyloric atresia (AR); 5. Muscular dystrophy, limb-girdle, autosomal recessive 17(AR) | phenotype mismatch |
| *EP300* | chr8:41574392 | NM_001429;exon31 | c.6677G>A (p.R2226Q) | het/- | 0.000077 | B | Uncertain | 1. Colorectal cancer, somatic; 2. Menke-Hennekam syndrome 2 (AD); 3. Rubinstein-Taybi syndrome 2 (AD) | phenotype mismatch |
| *JAK2* | chr9:5090811 | NM_004972;exon22 | c.2959G>A (p.E987K) | het/- | 0.000641 | D | Uncertain | 1. Erythrocytosis, somatic; 2. Leukemia, acute myeloid, somatic; 3. Myelofibrosis, somatic; 4. Polycythemia vera, somatic; 5. Thrombocythemia 3 ( AD, SMu); 6. Budd-Chiari syndrome, somatic | phenotype mismatch |
| *COL2A1* | chr12:48369150 | NM_001844;exon51 | c.3836C>T (p.P1279L) | het/- | - | D | Uncertain | 1. ?Epiphyseal dysplasia, multiple, with myopia and deafness; 2. ?Vitreoretinopathy with phalangeal epiphyseal dysplasia; 3. Achondrogenesis, type II or hypochondrogenesis; 4. Avascular necrosis of the femoral head; 5. Czech dysplasia; 6. Kniest dysplasia; 7. Legg-Calve-Perthes disease; 8. Osteoarthritis with mild chondrodysplasia; 9. Platyspondylic skeletal dysplasia, Torrance type; 10. SED congenital; 11. SMED Strudwick type; 12. Spondyloepiphyseal dysplasia, Stanescu type  13. Spondyloperipheral dysplasia; 14. Stickler syndrome, type I; 15. Stickler syndrome, type I, nonsyndromic ocular | phenotype mismatch |
| *LARS1* | chr5:145533344 | NM_020117;exon12 | c.1183G>A (p.D395N) | het/- | 0.0025641 | B | Uncertain | ?Infantile liver failure syndrome 1 (AR) | phenotype mismatch |
| *HOGA1* | chr10:99359522 | NM_138413;exon4 | c.554C>T (p.T185M) | het/- | 0.0053 | LD | Uncertain | Hyperoxaluria, primary, type III (AR) | phenotype mismatch |
| *TRPV4* | chr12:110252545 | NM_021625;exon2 | c.55_57delinsTCT (p.P19S) | het/- | - | - | Uncertain | 1. ?Avascular necrosis of femoral head, primary, 2(AD); 2. Brachyolmia type 3(AD); 3. Digital arthropathy-brachydactyly, familial(AD); 4. Hereditary motor and sensory neuropathy, type IIc(AD); 5. Metatropic dysplasia; 6. Neuronopathy, distal hereditary motor, type VIII; 7. Parastremmatic dwarfism; 8. Scapuloperoneal spinal muscular atrophy; 9. SED, Maroteaux type; 10. Spondylometaphyseal dysplasia, Kozlowski type; 11. Sodium serum level QTL 1 | phenotype mismatch |
| *COL3A1* | chr2:189849525 | NM_000090;exon2 | c.119C>T (p.A40V) | het/- | 0.0003 | B | Uncertain | 1. Ehlers-Danlos syndrome, vascular type(AD); 2. Polymicrogyria with or without vascular-type EDS(AR) | phenotype mismatch |
| *USH2A* | chr1:215847914 | NM_206933;exon63 | c.13339A>G (p.M4447V) | het/- | 0.0019231 | B | Uncertain | 1. Retinitis pigmentosa 39; 2. Usher syndrome, type 2A | phenotype mismatch |
| *USH2A* | chr1:216420119 | NM_206933;exon13 | c.2617G>A (p.G873R) | het/- | 0.000004 | LD | Uncertain | 1. Retinitis pigmentosa 39; 2. Usher syndrome, type 2A | phenotype mismatch |
| *SEC24D* | chr4:119736765 | NM_014822;exon5 | c.514C>G (p.P172A) | het/- | 0.0006418 | B | Uncertain | Cole-Carpenter syndrome 2(AR) | phenotype mismatch |
| *SEC24D* | chr4:119754754 | NM_014822;exon2 | c.98C>T (p.S33L) | het/- | 0.0001 | B | Uncertain | Cole-Carpenter syndrome 2(AR) | phenotype mismatch |
| *DCTN1* | chr2:74593745 | NM_004082;exon22 | c.2469A>T (p.V823=) | het/- | - | - | Uncertain | 1. Neuronopathy, distal hereditary motor, type VIIB(AD); 2. Perry syndrome (AD); 3. {Amyotrophic lateral sclerosis, susceptibility to}(AD,AR) | phenotype mismatch |
| *COL6A1* | chr21:47417338 | NM_001848;exon21 | c.1402G>A (p.E468K) | het/- | 0.000032 | LD | Uncertain | 1. Bethlem myopathy 1(AD,AR); 2. Ullrich congenital muscular dystrophy 1(AD,AR) | phenotype mismatch |
| *ABCA1* | chr9:107556793-107556797 | NM_005502;exon40 | c.5383-6_5383-3delTTTT  (splicing) | het/- | - | LD | Uncertain | 1.HDL deficiency, familial, 1(AR); 2.Tangier disease(AD) | phenotype mismatch |
| *PSMB9* | chr6:32825882 | NM_002800;exon4 | c.361G>C (p.A121P) | het/- | - | LD | Uncertain | ?Proteasome-associated autoinflammatory syndrome 3, digenic(AR) | phenotype mismatch |
| *CP* | chr3:148897398 | NM_000096;exon15 | c.2606C>G (p.T869R) | het/- | 0.0001 | B | Uncertain | 1. Cerebellar ataxia(AR); 2.Hemosiderosis, systemic, due to aceruloplasminemia(AR) | phenotype mismatch |
| *CLCN1* | chr7:143043294 | NM_000083;exon18 | c.2234A>G (p.N745S) | het/- | 0.0006524 | B | Uncertain | 1. Myotonia congenita, dominant(AR); 2. Myotonia congenita, dominant(AD). | phenotype mismatch |
| *TWNK* | chr10:102749493 | NM_021830;exon2 | c.1336A>G (p.I446V) | het/- | - | LD | Uncertain | 1. Mitochondrial DNA depletion syndrome 7 (hepatocerebral type) (AR); 2. Perrault syndrome 5 (AR); 3. Progressive external ophthalmoplegia with mitochondrial DNA deletions, autosomal dominant 3 (AD) | phenotype mismatch |
| *DNAH1* | chr3:52406398 | NM_015512;exon43 | c.6822C>G (p.D2274E) | het/- | 0.0018358 | B | Uncertain | 1. ?Ciliary dyskinesia, primary, 37 (AR);  2. Spermatogenic failure 18(AR) | phenotype mismatch |
| *WNT10A* | chr2:219754795 | NM_025216;exon3 | c.466G>T (p.A156S) | het/- | 0.000077 | B | Uncertain | 1. Odontoonychodermal dysplasia(AR); 2. Schopf-Schulz-Passarge syndrome(AR); 3. Tooth agenesis, selective, 4(AD, AR) | phenotype mismatch |
| *TTN* | chr2:179595873 | NM_133378;exon57 | c.13787C>A(p.A4596D) | het/- | 0.0006 | LD | Uncertain | 1.Cardiomyopathy, dilated, 1G(AD); 2.Cardiomyopathy, familial hypertrophic (AD); 3.Muscular dystrophy, limb-girdle, autosomal recessive 10 (AR); 4.Myopathy,myofibrillar, 9, with early respiratory failure(AD); 5.Salih myopathy(AR); 6.Tibial muscular dystrophy, tardive(AD) | phenotype mismatch |
| *TTN* | chr2:179426268 | NM_133378;exon275 | c.76887C>A(p.S25629R) | het/- | 0.0002243 | B | Uncertain |  | phenotype mismatch |
| *TTN* | chr2:179599611 | NM_133378;exon48 | c.11308A>T (p.T3770S) | het/- | 0.0018 | B | Uncertain |  | phenotype mismatch |
| *ATP2B2* | chr3:10452504 | NM_001683;exon3 | c.200-5C>G (splicing) | het/- | - | - | Uncertain | 1.Deafness, autosomal dominant 82 (AD); 2.Deafness, autosomal recessive 12, modifier of (AR) | phenotype mismatch |
| *PDE11A* | chr2:178576531 | NM_016953;exon13 | c.2119G>A (p.D707N) | het/- | 0.0007987 | D | Uncertain | Pigmented nodular adrenocortical disease, primary, 2 (AD) | phenotype mismatch |
| *COL6A3* | chr2:238243524 | NM_004369;exon41 | c.8974T>C (p.S2992P) | het/- | - | B | Uncertain | 1.Bethlem myopathy 1 (AD, AR); 2.Dystonia 27 (AR); 3.Ullrich congenital muscular dystrophy 1 (AD, AR) | phenotype mismatch |
| *RECQL4* | chr8:145740379 | NM_004260;exon9 | c.1561C>T (p.R521W) | het/- | 0.0003 | - | Uncertain | 1.Baller-Gerold syndrome (AR); 2.RAPADILINO syndrome (AR); 3.Rothmund-Thomson syndrome, type 2 (AR) | phenotype mismatch |
| *DIAPH3* | chr13:60240792 | NM_001042517;exon28 | c.3508C>G (p.L1170V) | het/- | - | B | Uncertain | Auditory neuropathy, autosomal dominant 1 (AD) | phenotype mismatch |
| *PRF1* | chr10:72357857 | NM_001083116;exon3 | c.1620A>G (p.Q540=) | het/- | 0.0135 | - | Uncertain | 1.Aplastic anemia; 2.Hemophagocytic lymphohistiocytosis, familial, 2 (AR); 3.Lymphoma, non-Hodgkin . | phenotype mismatch |
| *SYT2* | chr1:202573652 | NM_177402;exon3 | c.276G>T (p.K92N) | het/- | - | B | Uncertain | 1. Myasthenic syndrome, congenital, 7A, presynaptic, and distal motor neuropathy(AD); 2. Myasthenic syndrome, congenital, 7B, presynaptic (AR). | phenotype mismatch |
| *TNXB* | chr6:32029940 | NM_019105;exon20 | c.7162G>A (p.V2388M) | het/- | 0.0001118 | B | Uncertain | 1.Ehlers-Danlos syndrome, classic-like, 1 (AR); 2.Vesicoureteral reflux 8 (AD). | phenotype mismatch |
| *TNXB* | chr6:32036898 | NM_019105;exon16 | c.5603C>T (p.T1868M) | het/- | 0.0000319 | B | Uncertain |  | phenotype mismatch |
| *EDNRB* | chr13:78492734 | NM_001201397;exon2 | c.245G>A (p.R82Q) | het/- | 0.0122 | B | Uncertain | 1.?ABCD syndrome (AR); 2.Waardenburg syndrome, type 4A (AD, AR); 3. Hirschsprung disease, susceptibility to, 2 (AD) | phenotype mismatch |
| *CCM2* | chr7:45115522 | NM_031443;exon10 | c.1201A>G (p.N401D) | het/- | 0.0000544 | B | Uncertain | Cerebral cavernous malformations-2 (AD) | phenotype mismatch |
| *ZNF687* | chr1:151259519 | NM_020832;exon2 | c.752G>A (p.S251N) | het/- | 0.0007078 | B | Uncertain | Paget disease of bone 6 (AD) | phenotype mismatch |
| *ZNF750* | chr17:80789195 | NM_024702;exon2 | c.1136G>A (p.S379N) | het/- | 0.0000082 | B | Uncertain | Seborrhea-like dermatitis with psoriasiform elements | phenotype mismatch |
| *PCDH15* | chr10:55566517 | NM_001354429;exon37 | c.4979A>G (p.N1660S) | het/- | 0.0000556 | B | Uncertain | 1.Deafness, autosomal recessive 23 (AR); 2.Usher syndrome, type 1D/F digenic (AR, AD); 3.Usher syndrome, type 1F(AR) | phenotype mismatch |
| *NUP214* | chr9:134019967 | NM_005085;exon12 | c.1595C>T (p.P532L) | het/- | 0.0089744 | B | Uncertain | 1.Leukemia, acute myeloid, somatic; 2.Leukemia, T-cell acute lymphoblastic, somatic; 3.Encephalopathy, acute, infection-induced, susceptibility to, 9(AR) | phenotype mismatch |
| *NCF4* | chr22:37273779 | NM_000631;exon10 | c.934G>T (p.G312C) | het/- | 0.0089859 | B | Uncertain | Chronic granulomatous disease 3, autosomal recessive (AR) | phenotype mismatch |
| *SMARCAD1* | chr4:95155155 | NM_001128429;exon4 | c.419G>A (p.R140H) | het/- | 0.0005 | B | Uncertain | 1.Adermatoglyphia (AD); 2.Basan syndrome (AD); 3.Huriez syndrome(AD) | phenotype mismatch |
| *SLC9A3* | chr5:492130 | NM_004174;exon2 | c.268G>A (p.V90M) | het/- | 0.0124 | B | Uncertain | Diarrhea 8, secretory sodium, congenital (AR) | phenotype mismatch |
| *LRRK2* | chr12:40713845 | NM_198578;exon34 | c.4883G>C (p.R1628P) | het/- | 0.027635 | LD | Uncertain | Parkinson disease 8(AD) | phenotype mismatch |
| *SLC12A3* | chr16:56936319 | NM_000339;exon24 | c.2782C>T (p.R928C) | het/- | 0.0523163 | B | Uncertain | Gitelman syndrome (AR) | phenotype mismatch |

Het, Compound heterozygous; Hom, Homozygous; N/A, not applicable; D, damage; LD, likely damage; B, benign; AR, Autosomal recessive inheritance; AD, Autosomal dominant inheritance;

Data was analysed by from OMIM (https://www.omim.org/), Uniprot (https://www.uniprot.org/), MGI database (http://www.informatics.jax.org/), GnomAD https://gnomad.broadinstitute.org/), polyphen2 (http://genetics.bwh.harvard.edu/pph2/), SIFT/provean (http://provean.jcvi.org/index.php), MutationTaster (<https://www.mutationtaster.org/>), SpliceAI (<https://pypi.org/project/spliceai/1.2/>), CADD (https://cadd.gs.washington.edu/), GTEX (https://www.gtexportal.org/home/), Unique (https://rarechromo.org/), decipher (https://www.deciphergenomics.org/), Pubmed (<https://pubmed.ncbi.nlm.nih.gov/>).

**Reference**

[1] Verdura E, Rodriguez-Palmero A, Velez-Santamaria V, Planas-Serra L, de la Calle I, Raspall-Chaure M, Roubertie A, Benkirane M, Saettini F, Pavinato L et al: Biallelic PI4KA variants cause a novel neurodevelopmental syndrome with hypomyelinating leukodystrophy. Brain 2021, 144(9):2659-2669.

[2] Salter CG, Cai Y, Lo B, Helman G, Taylor H, McCartney A, Leslie JS, Accogli A, Zara F, Traverso M et al: Biallelic PI4KA variants cause neurological, intestinal and immunological disease. Brain 2021, 144(12):3597-3610.

[3]Pagnamenta AT, Howard MF, Wisniewski E, Popitsch N, Knight SJ, Keays DA, Quaghebeur G, Cox H, Cox P, Balla T et al: Germline recessive mutations in PI4KA are associated with perisylvian polymicrogyria, cerebellar hypoplasia and arthrogryposis. Hum Mol Genet 2015, 24(13):3732-3741.
